# Altered cytokine profiles in laminopathic patients

**DOI:** 10.1186/1750-1172-10-S2-O14

**Published:** 2015-11-11

**Authors:** Pia Bernasconi

**Affiliations:** 1 Neurology IV Unit - Neuroimmunology and Neuromuscular Diseases Unit, Foundation IRCCS Neurological Institute "Carlo Besta", Milan, Italy

## 

Prelamin A accumulation is known to dysregulate the NF-κB signaling cascade, causing a secretion of high levels of proinflammatory cytokines, which in turn might contribute to the pathologic aging observed in laminopathies, and in particular in HGPS[1]. In collaboration with researchers and clinicians of the Italian network for Laminopathies, we wondered whether it was possible to identify a pattern of cytokine expression that could discriminate laminopathy from other forms of muscular dystrophy and/or cardiomyopathy and a laminopathy with a cardiac involvement from one with only muscle involvement, with the final goal to identify biomarker(s) helpful for diagnosis, prognosis and evaluation of therapy efficacy. We analysed the cytokine profiles of sera collected from 37 patients affected by different forms of laminopathy (all *LMNA* mutations), 9 patients affected by genetically defined non-*LMNA* muscular dystrophy and 27 healthy individuals. Sera were screened for the expression levels of 16 cytokines, 6 chemokines, 5 growth factors and TGF-beta1, 2 and 3 by Luminex technology. Some pro-inflammatory cytokines were found to be differentially expressed in cardiopathic and non-cardiopathic patients compared to healthy controls, and among laminopathies with muscle and cardiac involvement, laminopathies without myopathy and muscular dystrophies. Interestingly, TGF-beta2 serum levels were higher in the *LMNA* patients than in healthy individuals and in patients with non*-LMNA* muscular dystrophy, suggesting a direct link between *LMNA* mutations and dysregulation of TGFbeta2 pathway, as indicated by previous and recent experimental studies[2,3].
